# Fluoroscopy-Guided Suture Anchor Placement Yields Excellent Accuracy for Arthroscopic Acetabular Labral Repair: A Cadaveric Study

**DOI:** 10.1016/j.asmr.2021.07.012

**Published:** 2021-08-19

**Authors:** Paul K. Herickhoff, Matthew Widner, Jason Mascoe, Wayne J. Sebastianelli

**Affiliations:** a Penn State Sports Medicine, State College, Pennsylvania, U.S.A.; b Penn State Orthopaedics and Rehabilitation, Hershey, Pennsylvania, U.S.A.

## Abstract

**Purpose:**

To determine the accuracy of fluoroscopy-guided suture anchor placement for arthroscopic acetabular labral repair in cadaveric hip specimens.

**Methods:**

Two sports medicine fellowship–trained surgeons performed arthroscopic hip surgery on 6 cadaveric specimens each. Suture anchors were placed at the 11-, 12-, 1-, 2-, 3-, and 4-o’clock positions of the acetabulum in each specimen using a previously described fluoroscopically guided technique. Gross dissection and thin-cut computed tomography scans were performed to assess for accuracy. The insertion angle between the subchondral bone and the drill bit immediately prior to suture anchor insertion was measured, and fluoroscopic visualization of the subchondral bone at each clock-face position was qualitatively graded as good, fair, or poor by 2 independent reviewers.

**Results:**

Overall, 90.3% of attempts (65 of 72) were entirely intraosseous, 5.5% (4 of 72) perforated the articular cartilage, and 4.2% (3 of 72) perforated the far cortex, rates that are comparable with those in previous cadaveric studies. There was no statistically significant difference in accuracy between the surgeons (*P* = .42) or between the various clock-face positions (*P* = .63). Neither the insertion angle (*P* = .26) nor visualization of the subchondral bone (*P* = .35) was significantly correlated with accuracy by gross dissection.

**Conclusions:**

In a cadaveric hip arthroscopy model, fluoroscopy-guided suture anchor placement yields excellent accuracy rates, similar to non–image-guided techniques.

**Clinical Relevance:**

Intra-articular suture anchor placement and intrapelvic suture anchor placement are known complications of arthroscopic acetabular labral repair. Fluoroscopically guided suture anchor placement can be a useful tool for hip arthroscopy surgeons performing acetabular labral repair and reconstruction, potentially reducing the risk of these complications.

Intra-articular suture anchor placement and intrapelvic suture anchor placement are known complications of arthroscopic acetabular labral repair.^1^^,^^2^ Technical tips to reduce the risk of these complications have been described, including using small-diameter and short suture anchors,^2^ using curved drill guides,^3^ performing acetabular rim trimming,^4^ starting 1 to 2.6 mm from the acetabular rim,^5^^,^^6^ and using distal portals.^2^^,^^7^^,^^8^ It has been recommended that the surgeon visualize the central compartment during drilling and anchor placement to ensure that the articular cartilage has not been violated^2^ and insert a nitinol wire into drilled holes to confirm they are contained prior to anchor insertion.^1^ However, even if errant suture anchor placement is avoided using these techniques, the articular cartilage or intrapelvic structures can still be damaged by inaccurate drilling.^9^

Fluoroscopy is routinely used during hip arthroscopy to confirm joint distraction and during initial portal placement. Recently, a fluoroscopically guided technique for suture anchor placement has been described that may reduce the risk of iatrogenic injury during acetabular labral repair^10^; however, this technique has not been validated. The purpose of this study was to determine the accuracy of fluoroscopy-guided suture anchor placement for arthroscopic acetabular labral repair in cadaveric hip specimens. Our hypothesis was that the fluoroscopically guided technique for suture anchor placement would have equal or better accuracy compared with previous studies.

## Methods

This study was approved by the institutional review board. Two sports medicine fellowship–trained, board-certified orthopaedic surgeons (P.K.H. and W.J.S.) performed arthroscopic hip surgery on 12 human fresh-frozen cadaveric hips without radiographic evidence of arthritis (Tönnis grade < 2) or hip dysplasia (lateral center-edge angle > 25°). The lateral center-edge angle was measured on post-procedure computed tomography (CT) scans^11^ and had a mean value of 43° (range, 33°-55°). Each surgeon operated on 3 left and 3 right hips. The sample size was based on data in previous studies.^6^^,^^12^^,^^13^ Eight cadaveric hips were matched pairs, and 4 were unmatched. Human cadaveric specimens were procured from Science Care (Phoenix, AZ). The average age of the specimens was 65.9 years (range, 39-99 years). Of the cadaveric hips, 10 were female hips and 2 were male hips.

Specimens were thawed to room temperature for 48 hours prior to arthroscopic surgery. The hip was secured to a mounting jig using a single one-quarter–inch carbon fiber drill bit through the shaft of the femur and 2 or 3 drill bits through the ilium. The hip was placed in the supine position with the femoral shaft perpendicular to the sourcil on anteroposterior fluoroscopic imaging. To simulate the clinical scenario of the C-arm being positioned between the legs, the C-arm base was positioned at a 45° angle relative to the femoral shaft in the horizontal plane. Traction was applied using the mounting jig. The central compartment was accessed via standard anterolateral (AL) and midanterior (MA) portals^14^ using the Seldinger technique. An interportal capsulotomy was created using a straight beaver blade (Samurai blade; Stryker, Greenwood Village, CO). A 50° radiofrequency ablator (SERFAS; Stryker) was used to mark the 12- and 3-o’clock positions on the labrum for reference, as described by Philippon et al.^15^ The radiofrequency ablator and a straight 4.0-mm shaver were used to expose the acetabular rim in preparation for suture anchor placement. No acetabular rim trimming or decortication of the rim was performed to eliminate this potentially confounding variable from affecting accuracy.^4^

Prior to suture anchor drilling, the surgeon directed the radiology technician to rotate the C-arm so that the x-ray beam was tangential to the clock-face position on the acetabular rim where the suture anchor would be placed, as has previously been described.^10^ The drill guide was positioned on the capsular side of the labral insertion, 2.3 to 2.6 mm from the acetabular rim. A 1.4-mm drill bit was drilled to a depth stop of 17 mm. Then, the suture anchor (Nanotack with length of 7.5 mm; Stryker) was inserted in a subcortical manner to the depth stop; the tabs were pulled, expanding the diameter of the anchor to 2.0 mm; and the sutures were cut. A straight drill guide was used for all insertion attempts but 1, in which a curved drill guide was used by surgeon 1 for a single 4-o’clock anchor. No redirecting was performed. This process was repeated at each clock-face position. All anchors were placed through the MA portal or AL portal at the surgeon’s discretion.

**Fig 1:**
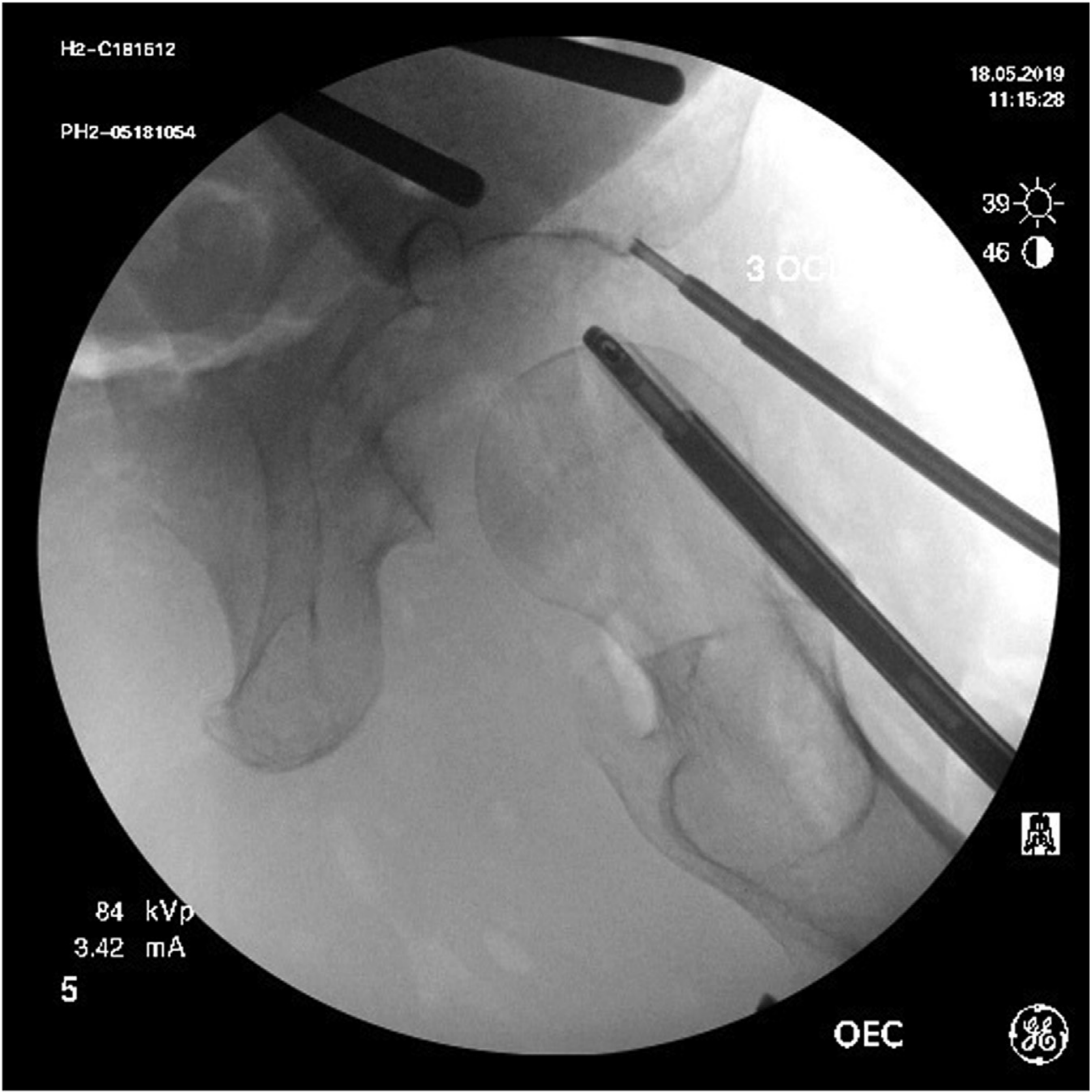
Tangential fluoroscopic image of subchondral bone of acetabulum at 3-o’clock position. The drill guide is in position for drilling of a 3-o’clock anchor.

**Fig 2:**
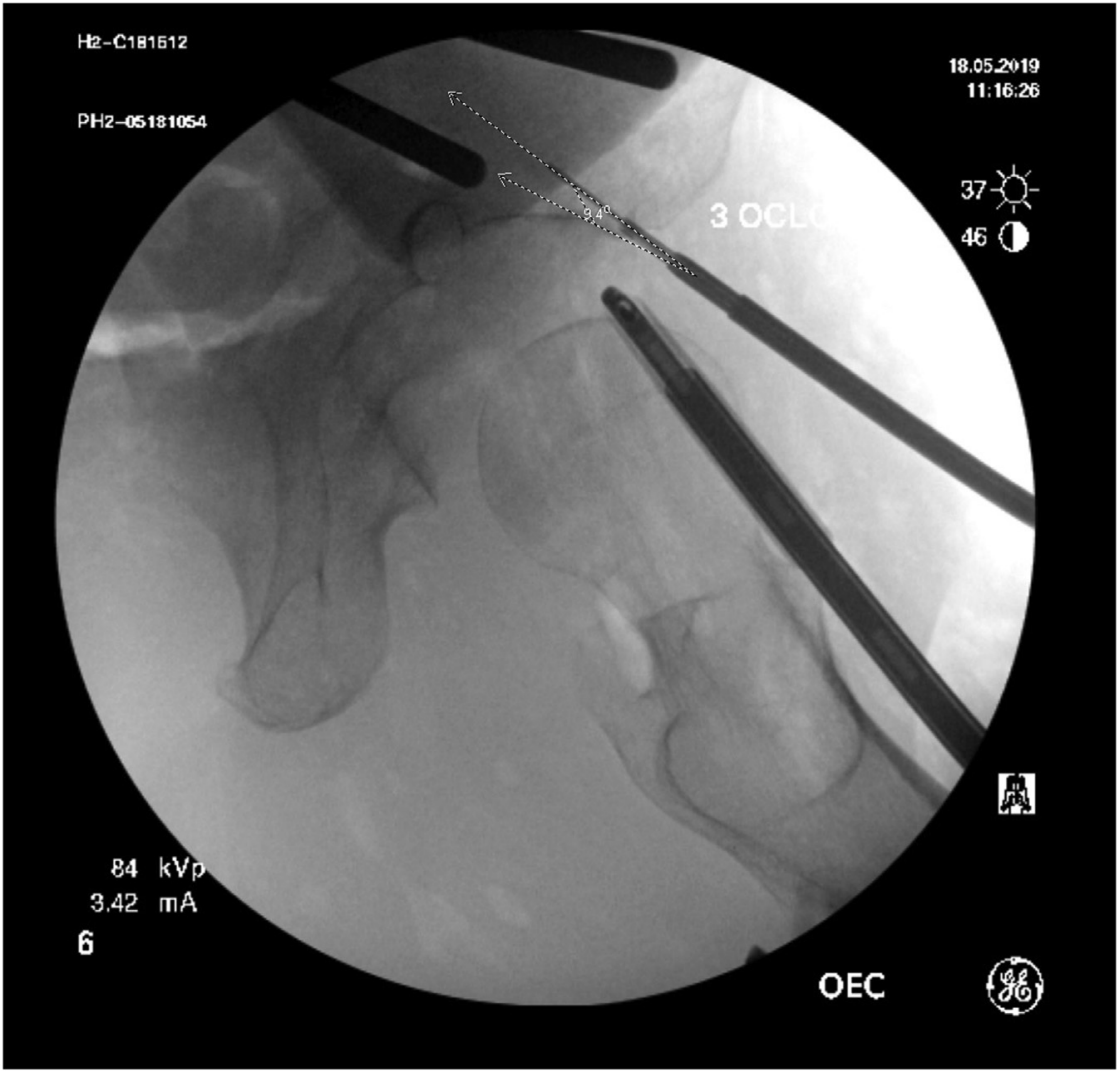
Tangential fluoroscopic image of subchondral bone at 3-o’clock position with drill bit in acetabular rim. The insertion angle, defined as the angle subtended by the drill bit and a line tangential to the subchondral bone, measures 9.4° on this image.

Two fluoroscopic images were saved to the picture archiving and communication system (IntelliSpace-Enterprise 4.4; Philips, Amsterdam, The Netherlands) at each clock-face position for each specimen: the first image prior to drilling (Fig 1) and the second image with the drill bit in the acetabulum (Fig 2). For the first set of images, visualization of the subchondral bone was qualitatively rated as good, fair, or poor at each clock-face position by 2 independent reviewers (PKH, MW) on 2 separate occasions at least 2 weeks apart. For the second set of images, the insertion angle, defined as the angle subtended by the drill bit and a line tangential to the subchondral bone (Fig 2), was measured by 2 reviewers (PKH, MW) on 2 separate occasions at least 2 weeks apart.

Postoperatively, thin-cut (1.5-mm) CT scans were obtained; gross dissection was then performed to assess for accuracy. Acetabular cartilage was not removed. The suture anchors were graded as being entirely intraosseous, perforating the articular cartilage, or perforating the far cortex (inner table of the pelvis).

Funding for this project was obtained from Penn State Health Milton S. Hershey Medical Center, Department of Orthopaedics and Rehabilitation, and Stryker Corporation, who provided the arthroscopy equipment and suture anchors.

### Statistical Analysis

Statistical analysis was performed using SAS statistical software (version 9.4; SAS Institute, Cary, NC). The Fisher exact test was performed to compare accuracy between the surgeons. The Kendall Tau-b test was used to compare accuracy by gross dissection to the divergence angle and visualization of the subchondral bone. The Cochran-Mantel-Haenszel test was performed to assess for differences in accuracy and visualization of the subchondral bone at the various clock-face positions. Intraclass and interclass correlation coefficients via a linear mixed-effects model and weighted κ values were calculated to assess intrarater and inter-rater agreement for the insertion angle and fluoroscopic visualization of the subchondral bone, respectively. Weighted κ values compared accuracy by gross dissection versus 1.5-mm thin-cut CT scans. Inter- and intraclass correlation coefficients and κ values were interpreted as follows: greater than 0.75, excellent; 0.40 to 0.75, fair to good; and less than 0.40, poor.^16^

## Results

Overall, 90.3% of attempts (65 of 72) were entirely intraosseous, 5.5% (4 of 72) perforated the articular cartilage, and 4.2% (3 of 72) perforated the far cortex by gross dissection (Table 1). There was no statistically significant difference in accuracy between the surgeons (*P* = .42).

**Table 1:** Individual and Combined Surgeon Accuracy of Suture Anchor Placement by Gross Dissection at 11- to 4-O’clock Positions on Acetabular Rim

	Accuracy, % (n)		Combined
	Surgeon 1	Surgeon 2	
Clock-face position			
11	100 (6 of 6)	100 (6 of 6)	100
12	83 (5 of 6, with 1 articular cartilage perforation)	83 (5 of 6, with 1 articular cartilage perforation)	83
1	100 (6 of 6)	83 (5 of 6, with 1 articular cartilage perforation)	92
2	100 (6 of 6)	100 (6 of 6)	100
3	67 (4 of 6, with 2 far cortex perforations)	100 (6 of 6)	83
4	67 (4 of 6, with 1 articular cartilage perforation and 1 far cortex perforation)	100 (6 of 6)	83
All positions	86 (31 of 36)	94 (34 of 36)	90 (65 of 72, with 4 articular cartilage perforations and 3 far cortex perforations)

Surgeon accuracy was not statistically different between the various clock-face positions (*P* = .63). There was no statistically significant difference (*P* = .99) in accuracy for suture anchors inserted from the AL portal (13 of 14 attempts [93%] entirely intraosseous) or MA portal (52 of 58 attempts [90%] entirely intraosseous). There was moderate correlation between accuracy by gross dissection and accuracy by CT scan (κ = 0.54).

Neither the insertion angle (*P* = .26) nor visualization of the subchondral bone (*P* = .35) was significantly correlated with accuracy by gross dissection. The combined qualitative ratings of visualization of the subchondral bone were statistically different between the clock-face positions (*P* < .0001), being worst at the 3- and 4-o’clock positions (Table 2).

**Table 2:** Combined Qualitative Ratings of Subchondral Bone Visualization at 11- to 4-O’clock Positions on Acetabular Rim (2 Instances Each for 2 Reviewers)

Clock-Face Position	Visualization of Subchondral Bone, n			
	Good	Fair	Poor	Total
11	25	21	2	48
12	40	8	0	48
1	40	8	0	48
2	34	12	2	48
3	14	19	15	48
4	10	22	16	48

The insertion angle showed good intrarater agreement (intraclass correlation coefficient 0.67) but poor inter-rater agreement (interclass correlation coefficient 0.26). Visualization of the subchondral bone showed excellent intrarater agreement (κ = 0.74) but poor inter-rater agreement (κ = 0.37).

## Discussion

The principal finding of this study is that fluoroscopically guided suture anchor placement into the acetabular rim from the 11-o’clock position to the 4-o’clock position was 90.3% accurate in a cadaveric hip arthroscopy model, with 5.5% of attempts perforating the articular cartilage and 4.2% of attempts perforating the far cortex or inner table of the pelvis. Our accuracy rate using this technique compares favorably with rates in previous studies. Dumont et al.^7^ used a Sawbones model (Vashon Island, WA) to determine the influence of curved versus straight drill guides, a drill starting point at the acetabular rim versus 2 mm off the rim, and drilling from the anterior, AL, or distal anterolateral accessory (DALA) portals on the rate of acetabular subchondral and far cortical perforation at the 11-, 12-, 1-, 2-, 3-, and 4-o’clock positions. They reported that, overall, 12.7% of drillings perforated the subchondral bone and 15% perforated the far cortex, with lower rates of perforation when using the DALA portal and starting 2 mm off the acetabular rim.

The largest cadaveric study assessing the safety of suture anchor insertion compared the rate of articular surface perforation and far cortical perforation using a straight drill guide from either the MA or DALA portal at the 9-, 11-, 12-, 1-, 2-, 3-, and 4-o’clock positions: Degen et al.^12^ reported no difference in the perforation rate between the MA and DALA portals and observed cumulative articular surface and far cortical perforation rates of 4.48% and 7.69%, respectively, with the highest risk of articular surface perforation at the 3-o’clock position. Our study found similar perforation rates to their study, although there was no difference in accuracy between the clock-face positions. Notable differences between their study and our study include that the rim was decorticated prior to drilling and suture anchor insertion in the study of Degen et al., which may increase accuracy by increasing the acetabular rim angle,^4^ and that we did not assess accuracy at the 9-o’clock position in our study because most hip arthroscopy beds do not permit fluoroscopic visualization of the rim at this position.^10^

A clear drawback of the fluoroscopically guided technique is the added radiation exposure to the surgeon and operating room staff.^10^ Therefore, in evaluating the risks and benefits to the patient and operating room staff, fluoroscopy-guided anchor placement is not necessary for all acetabular suture anchor insertions, and it may be best used by early adopters of hip arthroscopy or in situations in which the surgeon wants an additional check of the angle of the drill guide relative to the acetabular rim prior to drilling and placing the anchor.

Previous literature has offered technical tips and tricks to reduce the risk of iatrogenic injury during suture anchor placement for acetabular labral repair. Kelly et al.^17^ first described the surgical technique for arthroscopic acetabular labral repair, and they recommended a starting point more on the capsular side of the labrum than on the articular side to avoid penetration of the joint. Hernandez and McGrath^6^ performed the first anatomic study related to suture anchor placement, radially sectioning cadaveric acetabuli at the 12-o’clock, 1:30 clock-face, and 3-o’clock positions. Using the extracapsular insertion of the labrum as their starting point, they calculated a “safe angle” for suture anchor insertion of 20.1° to 27.6°. Consistent with these studies, our study used the extracapsular insertion of the labrum as the starting point for suture anchor drilling.

Matsuda et al.^2^ were the first authors to report on anchor-induced chondral damage in the hip. Several preventive techniques were recommended by these authors, including using shorter and smaller-diameter suture anchors, clearing the acetabular rim of capsule and synovium, and visualizing from the central compartment during drilling and suture anchor insertion.^2^ All 3 of these strategies were applied in our study.

Degen et al.^1^ reported the first cases of suture anchor penetration into the inner table of the pelvis, or psoas tunnel. To reduce the risk of this complication, they recommended inserting a nitinol wire into predrilled holes to ensure that the holes are contained and have not perforated the inner table of the pelvis.^1^ We agree with this recommendation in clinical practice; however, for this study, we did not check the drill holes prior to anchor insertion so that only 1 drill hole and suture anchor insertion attempt was assessed for accuracy at each clock-face position.

Curved drill guides have been recommended for acetabular labral repair to reduce the risk of iatrogenic damage to the articular cartilage.^3^ In a cadaveric study, Nho et al.^3^ measured the insertion angle as well as the distance from the tip of the suture anchor to the articular cartilage for suture anchors placed at the 1-, 2-, and 3-o’clock positions using either a straight or curved drill guide. They concluded that the curved drill guide increased the angle of insertion and the distance from the tip of the suture anchor to the articular cartilage at the 1-o’clock position but not at the 2- or 3-o’clock position on the acetabulum. In contrast, Dumont et al.^7^ found no difference in accuracy between curved and straight drill guides. The fluoroscopically guided technique assessed in our study is compatible with either straight or curved drill guides; however, because a straight drill guide was used for all suture anchor insertion attempts but 1, it is uncertain whether the use of curved drill guides would have an effect on surgeon accuracy with the fluoroscopically guided technique.

Several studies have investigated the influence of common hip arthroscopy portals on surgeon accuracy for suture anchor placement into the acetabular rim. In a cadaveric study, Stanton and Banffy^8^ placed pins into the acetabular rim from the 12- to 3-o’clock position through the AL, MA, and DALA portals. They concluded that the DALA portal allows pins to be placed at a greater distance from the articular surface than the MA and AL portals. Similarly, Dumont et al.^7^ concluded that the DALA portal yielded improved accuracy compared with the AL and anterior portals. In contrast, Degen et al.^12^ reported a decreased risk of intrapelvic or psoas tunnel perforation from the 2- to 4-o’clock position with anchor insertion from the MA portal compared with the DALA portal. In our investigation, all suture anchors at the 3- and 4-o’clock positions were inserted through the MA portal, and far cortical perforations (n = 3) only occurred at the 3- and 4-o’clock positions, consistent with the findings of Degen et al.^12^ Because suture anchors were inserted only through the MA and AL portals (with no difference in surgeon accuracy between these portals), no conclusion can be made regarding the effect of inserting suture anchors from the DALA portal on surgeon accuracy using the fluoroscopically guided technique.

The optimal angle for suture anchor insertion remains a subject of debate. Hernandez and McGrath^6^ recommended drilling at a 10° angle toward the joint surface, whereas other authors advised using distal portals and curved drill guides to increase the angle of insertion away from the joint.^2^^,^^3^^,^^8^ Although an increased insertion angle decreases the risk of articular surface perforation, it could also increase the risk of intrapelvic suture anchor placement, particularly where the acetabular rim is most narrow at the 3-o’clock position.^4^ In our study, insertion angle had no significant effect on surgeon accuracy; however, it should be noted that assessment of this was not the primary aim of this study, and the study was not adequately powered to assess this variable.

In our study, fluoroscopic visualization of the subchondral bone was significantly worse at the anterior acetabular rim (3- and 4-o’clock positions) compared with the superior acetabular rim. This difference could partially be explained by metal artifact from the mounting jig, affecting the contrast and resolution of the fluoroscopic images at the 3- and 4-o’clock positions. The quality of subchondral bone visualization had no effect on surgeon accuracy in this study, but again, the study was not adequately powered to answer this question.

Despite excellent and good intrarater agreement, there was poor inter-rater agreement on the quality of visualization of the subchondral bone and measurement of the insertion angle. Better delineation of where to draw the line tangent to the subchondral bone when measuring the insertion angle, as well as a more detailed description of the “good,” “fair,” and “poor” fluoroscopic visualization categories, may have improved our inter-rater reliability.

### Limitations

We acknowledge several limitations to our study. First, this study lacked a control group. Because of lack of funding, we were unable to acquire a sufficient number of cadavers for both control and investigational groups. Therefore, it is unknown whether the fluoroscopically guided technique resulted in any improvement in surgeon accuracy above and beyond the surgeons’ baseline accuracy using arthroscopy-only guided techniques. Second, there was only moderate correlation between the accuracy by gross dissection and the accuracy by thin-cut (1.5-mm) CT scan. The small sizes of the drill hole (1.4 mm) and anchor (2.0 mm) made both more difficult to visualize on the CT scan than expected. Although thinner CT cuts could have improved our visualization, we chose 1.5-mm cuts because this is standard clinical practice at our institution. Consistently with other cadaveric studies,^12^ however, gross dissection of the specimens was performed and was considered the gold standard. Third, the acetabular rim was not decorticated prior to suture anchor insertion, as is often performed in practice. Because Lertwanich et al.^4^ have shown that acetabular rim trimming significantly increases the safe angle for suture anchor insertion and because variability in the amount of rim decortication or trimming between surgeons and specimens was expected, we decided to eliminate rim trimming as a potential confounder of the data. As such, the accuracy rates reported in this study represent a worst-case scenario, and better accuracy rates are expected in cases in which rim trimming is performed in combination with the fluoroscopically guided technique. Fourth, a curved drill guide was used in only 1 of the 72 anchor insertion attempts, so the accuracy of the fluoroscopically guided technique with a curved drill guide is unknown. Fifth, none of the cadavers had coxa profunda, protrusio, or dysplasia, so accuracy rates with the fluoroscopically guided technique could vary in patients with abnormal acetabular morphology. Sixth, suture anchors were not inserted through the DALA portal, so the effect of using the DALA portal on the accuracy of the fluoroscopically guided technique for this portal is unknown. Comparing the accuracy of anchor insertion through the MA portal versus the DALA portal would have required several more cadavers (in matched hip pairs), similarly to the study by Degen et al.,^12^ and unfortunately, we did not have enough funding to obtain this quantity of cadavers. Finally, a single suture anchor system was used in this study, so these results may not be generalizable to other anchors used for acetabular labral repairs.

## Conclusions

In a cadaveric hip arthroscopy model, fluoroscopy-guided suture anchor placement yields excellent accuracy rates, similar to non–image-guided techniques.

## Supplementary Data

ICMJE author disclosure forms
